# A social differential outcomes learning task: Performance, EEG, and questionnaire data

**DOI:** 10.1016/j.dib.2020.106590

**Published:** 2020-11-26

**Authors:** Pierre Gander, Jonathan Rittmo, Rickard Carlsson, Robert Lowe

**Affiliations:** a Department of Applied Information Technology, University of Gothenburg, Gothenburg, Sweden; b School of Philosophy, Psychology and Language Sciences, University of Edinburgh, Edinburgh, UK

**Keywords:** Affective processing, Social learning, Inference, Knowledge transfer, EEG

## Abstract

This article contains performance data, questionnaire ratings, and EEG data from a differential outcomes learning task from two experiments. In both experiments, the standard differential outcomes learning task was extended to involve a social dimension, in order to capture how people can learn from others by observation. In Experiment 1 (*N* = 20), using a within-subjects design, participants learned pairings of image stimuli in four conditions: 1) individual-differential outcomes, 2) individual-non-differential outcomes, 3) social-differential outcomes, and 4) social-non-differential outcomes. The social condition had a screen-captured video recording of the outcomes (but not the actions themselves) of another person performing the task. During the task, the performance of the participants was measured. After the task, participants rated their experience in a questionnaire. The procedure for Experiment 2 (*N* = 33) was similar to Experiment 1, but with a stronger social manipulation using a video of another person's face showing facial expressions reflecting the outcomes. In addition, EEG was measured while performing the task. For more insight, please see Vicarious value learning: Knowledge transfer through affective processing on a social differential outcomes task (Rittmo et al., 2020).

## Specifications Table

**Table:** 

Subject	Experimental and Cognitive Psychology
Specific subject area	Differential outcomes learning
Type of data	Tables Questionnaires
How data were acquired	Experimentation software programmed in Java for stimulus presentation and response recording, running on laptop computer EEG recordings using OpenBCI headset Mark IV Survey using paper and pencil
Data format	Raw Cleaned Processed Analyzed
Parameters for data collection	The study had a 2 × 2 within-subjects experimental design with the measures: Performance on learning task, EEG measures, Questionnaire ratings of experiences during learning task
Description of data collection	Experiment 1: Participants’ learning on a memory task was recorded on a computer. Each participant did four tasks (order balanced between participants): 1) social-differential outcomes, 2) social-non-differential outcomes, 3) individual-differential outcomes, 4) individual-non-differential outcomes. The social condition had a recording of another person's behavioural outcomes on the screen (but not their actions). After the task, participants rated their experience in a questionnaire. Experiment 2: same as Experiment 1, but with a stronger social manipulation using a video of another person's face, and also measuring EEG during the task.
Data source location	Institution: Department of Applied Information Technology, University of Gothenburg City/Town/Region: Gothenburg Country: Sweden Latitude and longitude (GPS coordinates) for collected data: 57°42′23.9″N 11°56′13.7″E
Data accessibility	With the article
Related research article	Rittmo, J., Carlsson, R., Gander, P., & Lowe, R. (2020). Vicarious value learning: Knowledge transfer through affective processing on a social differential outcomes task. *Acta Psychologica, 209*, 1–14. https://doi.org/10.1016/j.actpsy.2020.103134. Article ref: ACTPSY_103134

## Value of the Data

• The data shows differential outcomes learning in a social situation, which is, to the authors’ knowledge the first study of differential outcomes training in a social context.
• The data can be used by experimental psychologists producing future investigations of differential outcomes training in social contexts; in particular results of first stage performance and third stage performance in the differential outcomes (experimental) conditions versus the non-differential outcomes (control) conditions might provide a useful comparison for future individual versus social experimental scenarios.
• The data can be compared for other differential outcomes tasks, e.g., using other stimuli material or other implementation of a social condition – is performance affected by specific types of stimuli? Perhaps video stimuli?
• The questionnaires can be used in future studies; these data can provide other comparisons for perception of vicarious experience of other's affective states (emotional empathy) or goals (cognitive empathy) in given studies. This might be useful to explain experimental data for similar social based differential outcomes studies.

## 1 Data Description

The data is from two experiments in which participants carry out an individual and a social differential outcomes task. From Experiment 1, there are three data files. One spreadsheet describes the learning performance (**exp1_performance_long_format.xlsx**). The spreadsheet lists the participant number (ID), whether the task was individual or social (type_task), whether it was a differential outcomes task (DO) or a non-differential outcomes task (NDO) (type_out), the stage: S1 or S3, and Number correct responses in last vs first block of 5 trials for Stage 1 and 3 respectively (Out_sum). Another spreadsheet contains the questionnaire data for Experiment 1 (**exp1_questionnaire.xlsx**), The file contains Gender (1 = Male, 2 = Female), Age, and Q1–Q11: Ratings for questions 1 to 11 using the scale 1 = strongly disagree, 2 = disagree, 3 = neutral, 4 = agree, 5 = strongly agree (note that participant number is not available for this data file due to clerical error). A copy of the questionnaire given to participants for Experiment 1 is also provided (**exp1_questionnaire_text.pdf**).

From Experiment 2, there are four data files. One spreadsheet contains the learning performance of the participants (**exp2_performance_long_format.xlsx**), listing Condition: 1 (Individual DOT), 2 (Individual NDOT), 3 (Social DOT), 4 (Social NDOT); Stage: 1 (Stage 1) or 3 (Stage 3); Out_sum_f5: Number of correct responses the in last block of 5 for Stage 1 and first block of 5 for Stage 3; Subject: Participant number; type_task: Social or Individual; type_out: DOT (differential outcomes) or NDOT (non-differential outcomes); Out_sum_tot: Total number of correct responses. Cleaned and filtered EEG data from Experiment 2 can be found in another spreadsheet (**exp2_eeg_long_format_8_12hz.xlsx**) with the contents Subject: Participant number; frequency: (the same for all); location: Location on scalp of electrodes in 10–20 system: Fp (average of Fp1 and Fp2), C (average of C3 and C4), P (average of P7 and P8), O (average of O1 and O2); condition: Individual (NSoc) or social (Soc); and power: amplitude in microvolt. The questionnaire data for Experiment 2 is in another spreadsheet (**exp2_questionnaire.xlsx**) which contains Age, Gender, Participant number, and the ratings of all 14 questions for each participant on a 7-point Likert scale (1 = strongly disagree, 7 = strongly agree). A copy of the questionnaire itself is also available (**exp2_questionnaire_text.pdf**).

## 2 Experimental Design, Materials and Methods

### 2.1 Experiment 1

#### 2.1.1 Participants

In order to estimate the sample size needed, an a-priori power analysis was performed with G*Power [1] using the effect size specification option “as in SPSS”, the effect size found specifically for the transfer of control procedure obtained via the meta-analysis by McCormack et al. [2], eta squared = 0.33, power = 0.8, and alpha = 0.05 resulted in *N* = 20. The participants were 9 male and 11 female students at the University of Gothenburg, with ages between 20 and 44 years (*M* = 27.74). They were rewarded with a cinema ticket for their participation.

#### 2.1.2 Design

The experiment was a 2 × 2 repeated measures design with the four conditions: i) Individual Differential Outcomes Training, ii) Individual Non-Differential Outcomes Training, iii) Social Differential Outcomes Training, iv) Social Non-Differential Outcomes Training. Thus, there are two independent variables with two levels each: i) Differential vs Non-Differential Outcomes training, ii) Individual vs Social scenario. Order effects were controlled so that social conditions came before and after Individual conditions; differential outcomes conditions came before and after non-differential outcomes conditions. Each condition consisted of a transfer of control procedure consisting of 3 stages of 20 trials each. The dependent variable was the number of correct responses in Stage 3 (calculated for the first 5 trials). (For the theory underlying the design, see Lowe et al. [3] and for interpretation of the data, see Rittmo et al. [4].)

#### 2.1.3 Materials and apparatus

Each participant carried out every condition on a computer setup where they observed the presentation of stimuli and outcomes on a monitor and produced mouse click responses. Images for the stimuli were taken from the Snodgrass standardised image set [5]. For each condition, six images were used (giving a total of 24 for all four conditions). Audio feedback was given for correct and incorrect responses. Sounds were taken from IADS (International Affective Digitized Sounds) [6] – positive and moderately positive for correct feedback, negative for incorrect feedback.

#### 2.1.4 Procedure

Participants were given a pre-experiment warm-up phase with stimuli different from the main experiment. Instructions were given orally before the experiment and written prompts were provided periodically on the monitor. Participants were informed that they would receive a cinema ticket for participation and an additional ticket as reward if they achieved a sufficiently high score. All stimuli images presented to the participants were presented randomly, one per trial, but such that there was always an equal number of each stimulus presented per stage. In Stage 1, Instrumental learning phase, participants were required to learn to associate 2 different stimuli images with appropriate responses in order to get either a high rewarding or low rewarding outcome (differential outcomes condition) or randomly either high or low reward (non-differential outcomes condition). Incorrect answers received a punishment (‘money’ loss). In Stage 2, Pavlovian learning phase, participants were required to learn to associate 4 new stimuli images with high or low rewarding outcomes (the stimuli predicted the outcomes in the differential outcomes condition but not in the non-differential outcomes condition). In the social condition, participants viewed a screen-capture (audio and video) of another person's performance on a different monitor and were told to observe and learn from the other's stimulus-outcome results. In Stage 3, Instrumental transfer test phase, participants again were required to associate images (stimuli) with responses. But now it was required to associate the four images used in Stage 2 to the response options in Stage 1. Note that this constituted a new set of associations for the participants to learn. After the experiment, participants completed a questionnaire measuring emotional engagement and experienced presence in the social condition (one participant failed to complete the questionnaire and was excluded). The questionnaire items were developed for this data collection (Cronbach's alpha = 0.83). (More details about the procedure can be found in Rittmo et al. [4].)

### 2.2 Experiment 2

#### 2.2.1 Participants

An a-priori power calculation was performed to estimate sufficient sample size for Experiment 2. The effect size of the differential and non-differential outcomes within the social level from Experiment 1 yielded Cohen's *d* = 0.47. Using this effect size, with power = 0.8 and alpha = 0.05 for a one-sided *t*-test (directional hypothesis), resulted in *N* = 29. Due to potential problems with the EEG equipment use and analysis, another four participants were added. Therefore, 33 students participated, all from the University of Gothenburg, and in each of the four conditions of Experiment 2 for the reward of a cinema ticket. The participants were aged between 22 and 46 years (*M* = 26.6) with 23 males and 10 females. One participant was excluded from the EEG analysis due to highly unreliable data not salvageable through the standardised processing steps used for all other participants.

#### 2.2.2 Design

Study 2 had the same design as Study 1 with the addition of EEG measures. The dependent variable for the EEG activity was power spectral density over the mu frequency band, extracted using fast Fourier transform. That is amplitude in the 8–12 Hz frequency obtained from C3 and C4, which is the typical site for mu detection since these locations are positioned over the motor cortex. Additionally, alpha band power over the frequencies 8–12 Hz for all remaining electrodes was obtained and averaged.

#### 2.2.3 Materials and apparatus

The materials for Experiment 2 were the same as for Experiment 1 with the exception of the stimuli in the social condition and an updated questionnaire. In Stage 2 (Pavlovian phase), the participant was shown a video of either the face of another person (confederate) playing the game (social condition), or an animation (non-social condition). The purpose of the animation was to have a comparably complex and informative stimulus, but that would not be perceived as social and yet would keep participants focused. The animation showed a randomly moving shape where the outcome images of Stage 1 were shown (faded in) at outcome presentation. Thus, the non-social video provided information for the participants to be able to carry out the stimulus-outcome pairing Stage of the experiment.

#### 2.2.4 Objective measure of confederate emotional expression

Two persons were used to play the role of confederate during the Pavlovian phase for the social conditions. One was used in the pre-experiment warm-up phase and another in the main experiment. Each confederate was instructed to express happiness when receiving the high reward and mild frustration when receiving the low reward. As a means of assessing objectively differential facial expressions we used the Noldus software FaceReader 8.0. An independent samples *t*-test revealed a statistically significant difference between low reward (including negative reward) vs high reward facial expressions, 95% CI[−.86, −.43], t(18) = −6.36, *p* < .001.

#### 2.2.5 Electroencephalogram recordings

The OpenBCI Cyton board was used to measure the electroencephalogram (EEG). The Cyton board is an 8-channel neural interface with a sample frequency of 250 Hz. Since the present study did not analyze higher frequencies than 25 Hz, the sampling rate was deemed sufficient since only a sampling rate of 2.5 times that of the frequency is required for analysis [7]. The communication is wireless via Bluetooth to a computer and OpenBCI's own graphical user interface was used for the recording of the data. Further, the associated OpenBCI headset Mark IV was used. The headset is able to target 35 electrode locations of the 10–20 system. The locations used in Experiment 2 are those of the original locations of the headset. After a pilot study using the device the original electrode placements were shown to generate superior contact compared to other locations and provided a coarse distributed signal across the scalp. Electrodes at the earlobes were used as reference. The locations used were: Fp1, Fp2, C3, C4, P7, P8, O1 and O2 according to the 10–20 system. Fp1, Fp2, O1 and O2 have previously been used for emotional detection [8] and C3 and C4 are common locations for detecting mu rhythmicity [9] the suppression in activity of which (in these central brain regions) being considered to reflect mirror neuron activity [9]. It should be noted though that the robustness of mu suppression as an indicator for mirror neuron system activation has been questioned [10].

#### 2.2.6 Procedure

The procedure for Experiment 2 was similar to that of Experiment 1, with the difference that participants now wore the EEG headset. The main task-based difference between Experiment 1 and Experiment 2 was the use of videos (visual) without audio in the Pavlovian phase (Stage 2) of the experiment. The social video was designed to: a) increase the sense of social presence, b) allow for emotional expressions to provide a means for vicarious value learning.

## Ethics Statement

Written informed consent was obtained for experimentation with human subjects.

## Declaration of Competing Interest

The authors declare that they have no known competing financial interests or personal relationships which have, or could be perceived to have, influenced the work reported in this article.

## Appendix Supplementary materials

Image, application 1
