# Supplementary material for: A social differential outcomes learning task: Performance, EEG, and questionnaire data
Source: Data Brief. 2020 Nov 26;33:106590. doi: 10.1016/j.dib.2020.106590 (PMC7726659; doi:10.1016/j.dib.2020.106590)
Supplement: Supplementary file 1 [file mmc1.zip › Data_in_brief_revised/exp2_questionnaire_text.pdf]

Experiment questionnaire

*Obligatorisk

1. Age *

2. Gender *

Markera endast en oval.

- Man
- Woman
- Other

Please circle your level of agreement with the following questions.

1-7 corresponds to: Strongly Disagree, Disagree, Neutral, Agree, Strongly agree

3. 1. I understood which options were the most rewarding *

Markera endast en oval.

	1	2	3	4	5	6	7	
Strongly disagree								Strongly agree

4. 2. I found the task engaging *

Markera endast en oval.

	1	2	3	4	5	6	7	
Strongly disagree								Strongly agreee

5. 3. I experienced anxiety while performing the task *

Markera endast en oval.

	1	2	3	4	5	6	7	
Strongly disagree								Strongly agreee

6. 4. I experienced frustration while performing the task *

Markera endast en oval.

	1	2	3	4	5	6	7	
Strongly disagree	◯	◯	◯	◯	◯	◯	◯	Strongly agreee

7. 5. I experienced excitement while performing the task *

Markera endast en oval.

	1	2	3	4	5	6	7	
Strongly disagree	◯	◯	◯	◯	◯	◯	◯	Strongly agreee

8. 6. I experienced happiness while performing the task *

Markera endast en oval.

	1	2	3	4	5	6	7	
Strongly disagree	◯	◯	◯	◯	◯	◯	◯	Strongly agreee

9. 7. I experienced that the other participant was involved in the activity *

Markera endast en oval.

	1	2	3	4	5	6	7	
Strongly disagree	⬭	⬭	⬭	⬭	⬭	⬭	⬭	Strongly agreee

10. 8. I understood what the other participant was doing *

Markera endast en oval.

	1	2	3	4	5	6	7	
Strongly disagree	⬭	⬭	⬭	⬭	⬭	⬭	⬭	Strongly agreee

11. 9. I did not experience the other participant’s goals *

Markera endast en oval.

	1	2	3	4	5	6	7	
Strongly disagree	⬭	⬭	⬭	⬭	⬭	⬭	⬭	Strongly agreee

12. 10. I recognized what the other participant felt *

Markera endast en oval.

	1	2	3	4	5	6	7	
Strongly disagree	◯	◯	◯	◯	◯	◯	◯	Strongly agreee

13. 11. I experienced the other participant’s emotions *

Markera endast en oval.

	1	2	3	4	5	6	7	
Strongly disagree	◯	◯	◯	◯	◯	◯	◯	Strongly agreee

14. 12. I did not experience the animation as an active agent *

Markera endast en oval.

	1	2	3	4	5	6	7	
Strongly disagree	◯	◯	◯	◯	◯	◯	◯	Strongly agreee

15. 13. I could draw the connections between what occurred in the animation and the pictures presented *

Markera endast en oval.

	1	2	3	4	5	6	7	
Strongly disagree	◯	◯	◯	◯	◯	◯	◯	Strongly agreee

16. 14. I could draw the connections between the other participant's reactions and the pictures presented *

Markera endast en oval.

	1	2	3	4	5	6	7	
Strongly disagree	◯	◯	◯	◯	◯	◯	◯	Strongly agreee

17. If you wish to receive feedback about the results of the study, please fill in your email address

____

Filled in by experiment leader

18. Subject number *

____

19. Order *

Markera endast en oval.

- 1
- 3
- 5
- 7

Det här innehållet har varken skapats eller godkänts av Google.

Google Formulär
