# Supplementary material for: A social differential outcomes learning task: Performance, EEG, and questionnaire data
Source: Data Brief. 2020 Nov 26;33:106590. doi: 10.1016/j.dib.2020.106590 (PMC7726659; doi:10.1016/j.dib.2020.106590)
Supplement: Supplementary file 1 [file mmc1.zip › Data_in_brief_revised/exp1_questionnaire_text.pdf]

We have a few questions for you

Please circle your level of agreement with the following questions.

I understood which options were the most rewarding

strongly disagree	disagree	neutral	agree	strongly agree
○	○	○	○	○

I found the game engaging

strongly disagree	disagree	neutral	agree	strongly agree
○	○	○	○	○

I experienced anxiety while playing the game

strongly disagree	disagree	neutral	agree	strongly agree
○	○	○	○	○

I experienced frustration while playing the game

strongly disagree	disagree	neutral	agree	strongly agree
○	○	○	○	○

I experienced excitement while playing the game

strongly disagree	disagree	neutral	agree	strongly agree
○	○	○	○	○

I experienced happiness while playing the game

strongly disagree	disagree	neutral	agree	strongly agree
○	○	○	○	○

Please state your gender

male	female
○	○

Please state your age

____

Please answer some questions about the other person playing the game.

I experienced that the other person participated in the activity

strongly disagree	disagree	neutral	agree	strongly agree
○	○	○	○	○

I understood what the other person was doing

strongly disagree	disagree	neutral	agree	strongly agree
○	○	○	○	○

I experienced the other person's goals

strongly disagree	disagree	neutral	agree	strongly agree
○	○	○	○	○

I knew what the other person felt

strongly disagree	disagree	neutral	agree	strongly agree
○	○	○	○	○

I experienced the other person's emotions

strongly disagree	disagree	neutral	agree	strongly agree
○	○	○	○	○
